# New Appliances and Things Medical

**Published:** 1904-06-04

**Authors:** 


### NEW APPLIANCES AND THINGS MEDICAL.

[We shall be glad to receive at our Office, 28 & 29 Southampton Street, Strand, London, W.C., from the manufacturers, specimens of all new preparations and appliances which may be brought out from time to time.]

### WHITE SEAL WHISKY.

(HENRY SIMPSON AND COMPANY, 6 CROSBY SQUARE, LONDON, E.C.)

THE whisky under consideration has a good, full, mellow flavour, and, so far as taste is concerned, has all the characteristics of a genuine, well-matured whisky. It is a blended whisky, and is stated to be chiefly composed of pot-still whiskies, there being present a small quantity of Scotch grain, which is thought to make the product more palatable and more digestible. The chemical examination which we have made of it shows that it contains an average quantity of furfural, a substance usually held to be characteristic of genuine pot-still whisky, or at any rate, of a whisky, the chief constituent of which is genuine pot-still whisky as distinguished from silent spirit. Dietetically, we have every reason to believe that the whisky is a sound product and to be recommended.

### FUSSELL'S EVAPORATED CREAM.

IN our notice last week of Messrs. Fussell and Co.'s Evaporated Cream the name of the article appeared through a printer's error as "Silon Butterfly Brand." The correct name is "Silver Butterfly Brand."

